# Consistent Measurement of Parasite-Specific Antigen Levels in Sera of Patients with Neurocysticercosis Using Two Different Monoclonal Antibody (mAb)-Based Enzyme-Linked Immunosorbent Assays

**DOI:** 10.3390/pathogens12040566

**Published:** 2023-04-06

**Authors:** Yesenia Castillo, Luz M. Toribio, Carolina Guzman, Gianfranco Arroyo, Cindy Espinoza, Herbert Saavedra, Javier A. Bustos, Pierre Dorny, Seth E. O’Neal, Hector H. Garcia

**Affiliations:** 1 Center for Global Health, Universidad Peruana Cayetano Heredia, Lima 15202, Peru; yesenia.castillo.b@upch.pe (Y.C.);; 2 Cysticercosis Unit, National Institute of Neurological Sciences, Lima 15030, Peru; 3 Department of Biomedical Sciences, Institute of Tropical Medicine, 2060 Antwerp, Belgium; 4 School of Public Health, Oregon Health & Sciences, Portland State University, Portland, OR 97207, USA; 5 Department of International Health, Bloomberg School for Public Health, Johns Hopkins University, Baltimore, MD 21205, USA

**Keywords:** neurocysticercosis, *Taenia solium*, Ag-ELISA, monoclonal antibodies, agreement

## Abstract

Monoclonal antibody (mAb)-based enzyme-linked immunosorbent assay (ELISA) is a complementary diagnosis technique for neurocysticercosis (NCC), which detects circulating parasite antigen (Ag) indicative of viable infection and Ag levels that correlate well with the parasite burden. In this study, we compared the performance of two Ag-ELISA techniques for the detection of NCC. We assessed the agreement between our in-house TsW8/TsW5 Ag-ELISA and the widely used B158/B60 Ag-ELISA for measuring *T. solium* antigen levels in the sera from 113 patients with calcified, parenchymal, and subarachnoid NCC. Concordance was demonstrated evaluating the limits of agreement (LoAs) stratified by the type of NCC. Both ELISA’s detected 47/48 (97.8%) subarachnoid NCC cases. In parenchymal and calcified NCC, the B158/B60 Ag-ELISA detected 19/24 (79.2%) and 18/41 (43.9%) cases, while the TsW8/TsW5 Ag-ELISA detected 21/24 (87.5%) and 13/41 (31.7%), respectively. Parenchymal and calcified NCC obtained a perfect agreement (100%), indicating that all sample results were within the predicted LoA, while for subarachnoid NCC, the agreement was 89.6%. The high concordance between the assays was confirmed by Lin’s concordance coefficient (LCC = 0.97). Patients with viable parenchymal NCC (LCC = 0.95) obtained the highest concordance between assays, followed by subarachnoid NCC (LCC = 0.93) and calcified NCC (LCC = 0.92). The TsW8/TsW5 Ag-ELISA and B158/B60 Ag-ELISA showed high Ag measurement correlations across diverse types of NCC.

## 1. Introduction

Neurocysticercosis (NCC), a parasitic infection of the human central nervous system (CNS), is caused by the accidental ingestion of *Taenia solium* eggs through fecal–oral contamination [1,2]. NCC is considered a neglected disease and the most important cause of acquired epilepsy in most of the world. It represents a public health problem due to the burden of neurological disease it causes, resulting in significant morbidity and mortality, as well as high hospitalization costs [3]. This parasitosis is endemic not only in developing countries where the sanitation measures are inadequate, but it is also present in industrialized countries with high immigration rates [4,5,6,7].

A highly heterogeneous clinical presentation and the lack of specific symptoms makes diagnosis of NCC a challenge [8]. In clinical settings, the definitive diagnosis of NCC is provided by neuroimaging findings using either a computed tomography (CT) scan or magnetic resonance imaging (MRI), which allow the visualization of the parasites in the CNS [9]. However, neuroimaging findings are not always conclusive, as some lesions are nonspecific, while others may be missed on CT or MRI [10,11]. Neuroimaging equipment is also frequently not available in clinical settings in most rural endemic areas. In these scenarios, immunodiagnostic tools can contribute to the diagnosis of NCC and guide the management of patients, including decisions to transfer them to a more equipped clinical setting for specialized care [12,13].

The reference immunoassay for NCC diagnosis is the enzyme-linked immunoelectrotransfer blot using lentil lectin purified parasite antigen (LLGP-EITB) for the detection of antibodies against 7 *T. solium* glycoproteins, which an estimated sensitivity of 98% in individuals with more than 1 viable brain lesion, and no cross-reactions in clinical settings [14]. However, antibody detection on EITB does not discriminate the presence of viable infection since antibodies may persist long after cyst resolution or result from exposure only or aborted infections [15,16]. Assays to detect circulating parasite antigens have the advantage of demonstrating the presence of viable infection and may correlate with the parasite burden and severity [17,18]. Further, antigen levels drop immediately after cysticidal treatment, for which antigen detection is preferred to monitor the efficacy of treatment in NCC patients [19,20].

The development of monoclonal antibodies (mAbs) and their adaptation in sandwich enzyme-linked immunosorbent assay (Ag-ELISA) formats for antigen detection greatly improved their utility for NCC diagnosis. The only two reported *T. solium* Ag-ELISA’s, HP10 and B158/B60, use two sets of mAbs initially developed against *Taenia saginata* cysticercal antigens, and were later adapted for use in human NCC because of its cross-reaction with *T. solium* cysticercal antigens, showing acceptable levels of sensitivity and specificity, respectively [21,22]. One of these assays, the B158/B60 Ag-ELISA, is commercially available (Advanced Practical Diagnostics “apDia”) [23] and is the most widely used assay for antigen detection in NCC in different studies. However, the sensitivity of this assay decreases in NCC cases with few brain cysts [24,25]. Our group has produced a set of 21 specific anti-*T. solium* mAbs reacting against antigens of the whole cyst, vesicular fluid, and secretory/excretory products. Eight of these mAbs were able to detect parasite antigens in serum and urine samples from patients with NCC [26], of which the most promising pair of mAbs, TsW8 (capture antibody) and TsW5 (biotin-labelled secondary antibody), were selected and adapted to a sandwich Ag-ELISA format. Here, we report the use of our in-house TsW8/TsW5 Ag-ELISA for antigen detection in NCC and assess its level of agreement with the widely used B158/B60 Ag-ELISA in a group of defined serum samples from NCC patients.

## 2. Materials and Methods

### 2.1. Study Design and Participants

This study assessed the agreement between our in-house TsW8/TsW5 Ag-ELISA and the B158/B60 Ag-ELISA for measuring antigen levels in 113 archive serum samples from patients with NCC, consecutively enrolled in a cohort study between September 2018 and January 2019 at the Instituto Nacional de Ciencias Neurologicas (INCN) in Lima, Peru. Participants were eligible if they had one or more cysticercotic lesions determined on CT scan or MRI and if they were also positive on LLGP-EITB for antibody detection (one or more bands). Radiological information was obtained from each participant, and it included the location of each cyst in the CNS (parenchymal or subarachnoid), as well as the stage of parenchymal cysts (viable, degenerating, or calcified). A small group of 8 patients with ventricular NCC were excluded to keep NCC patients categorized as either subarachnoid or intraparenchymal NCC cases.

### 2.2. Ethics Statement

The anonymized samples used were collected under a prior study registered and approved by the main IRB of the Instituto Nacional de Ciencias Neurologicas (approval 031-2022-RNV-CIEI-INCN), after obtaining written informed consent specifically authorizing the future use of samples for diagnostic studies.

### 2.3. Laboratory Procedures

All ELISA procedures were performed blindly by assigning a numeric code to each sample. The laboratory staff was also blind to the neuroimaging findings of patients. Sera were processed in duplicate, and discordant results (samples with 10% or more variation between duplicates) and borderline samples were reprocessed. Each Ag-ELISA plate included two pools from defined positive sera and eight negative control samples.

### 2.4. TsW8/TsW5 Ag-ELISA

Briefly, flat-bottom MAXISORP plates were coated with purified capture antibodies TsW8 diluted in carbonate bicarbonate buffer, pH 9.3–9.9, and were kept in incubation for 30 min at 37 °C while constantly moving. Plates were manually washed once with 180 µL of PBS Tween 0.05%, and then a blocking buffer (skimmed milk 1% diluted in PBS-Tween 0.05%) was added to each well and incubated for 30 min at 37 °C. The blocking buffer was discarded, and 100 µL of serum samples at 1:10 dilution, previously treated with trichloroacetic acid (TCA) at 5% to prevent a non-specific reaction caused by human glycoproteins or albumin in serum, were added to each well and incubated for another 30 min. After five consecutive washing steps, the biotin-labeled secondary antibody TsW5 was added. Peroxidase streptavidin diluted at 1:10,000 was subsequently added to recognize the secondary antibody, and the reaction was developed using an O-phenylenediamine (OPD) solution. The reaction was stopped with H_2_SO_4_ (2N), and ELISA plates were read in a spectrophotometer (SpectraMax ABS Molecular Devices LLC; San Jose, CA, USA) at 490/650 nm of wavelength to obtain optical density (OD) values. We divided the OD values by the average OD value from eight negative sera (‘cutoff’) to obtain the antigen ratios (expressed as continuous variables). We arbitrarily considered a positive Ag—ELISA result if the antigen ratio was ≥1.

### 2.5. B158/B60 Ag-ELISA

We performed the B158/B60 Ag-ELISA following the original methodology by Brandt et al., with the modifications for cysticercosis diagnosis as published by van Kerckhoven et al. [27]. Briefly, MAXISORP plates were coated with capture antibodies B158C11A10 and were kept in incubation for 30 min at 37 °C on a shaking plate. Plates were manually washed with PBS—Tween 0.05%, and blocking solution was added to each well. A total of 0.1 mL of serum samples, previously treated with TCA 5% (1:4 dilutions), were added to each well, and the plates were incubated for 15 min at 37 °C while constantly moving. After the washing steps, biotin-labeled B60H8A4 antibodies and streptavidin-HRP diluted 1:10,000 were used for detection. The reaction was developed, and the ELISA plates were read and interpreted as previously described.

### 2.6. Statistical Analysis

Patients’ demographic data (age in years and sex), serological outcomes (number of EITB antibody bands, Ag-ELISA ODs, OD ratios, and positive results), and neuroimaging findings (type of NCC: calcified parenchymal, viable parenchymal, and subarachnoid) were described using summary statistics. We compared demographic data and serological profiles by the type of NCC using bivariate tests. We used raw ODs for the agreement analysis of antigen levels between Ag-ELISA’s, since OD ratios can be systematically different between assays due to intrinsic differences in the OD readings of negative controls (used to obtain the OD ratios in each assay). ODs were transformed to a logarithm scale to approximate these values to the normal distribution and to determine additional limits of agreement between assays. The limits-of-agreement (LoAs) procedure between Ag-ELISAs was performed using Bland–Altman (BA) plots, accounting for proportional bias and heteroscedasticity. Lin’s Concordance Coefficient (LCC) was also calculated to determine the agreement between our TsW8/TsW5 Ag-ELISA and the reference B158/B60 Ag-ELISA for measuring antigen levels (log-ODs). All the analyses considered the total number of patients and were also stratified by the type of NCC. Statistical analysis was carried out in Stata/SE 17.0 (Stata Corp., College Station, TX, USA), using a significance level set to 5%.

## 3. Results

### 3.1. Characteristics of Study Participants and Comparisons by Type of NCC

Samples from 113 patients with NCC were included in the study, of which 41 (36.3%) had calcified parenchymal NCC, 24 (21.2%) had viable parenchymal NCC, and 48 (42.5%) had subarachnoid NCC. Patients had a mean age of 43.4 ± 14.9 years, and 57 (50.4%) were female and had an overall median of 3 EITB antibody bands (interquartile range (IQR): 3–7) (Table 1). We found statistical differences in the age of the patients by type of NCC. Cases with subarachnoid NCC (46.1 ± 14.3 years) and calcified parenchymal NCC (46.0 ± 15.5 years) were older than those with viable parenchymal NCC (33.5 ± 10.7 years, *p* = 0.009). On the contrary, differences in the sex of cases were not statistically different by the type of NCC. A stronger antibody response on EITB was observed in patients with subarachnoid NCC (median of EITB bands: 7 [IQR: 5–7]) compared to the antibody responses in patients with viable parenchymal NCC (median of EITB bands: 3 [IQR: 2–5]) and patients with calcified parenchymal NCC (median of EITB bands: 3 [IQR: 2–3]; *p* < 0.001).

**Figure 1:**
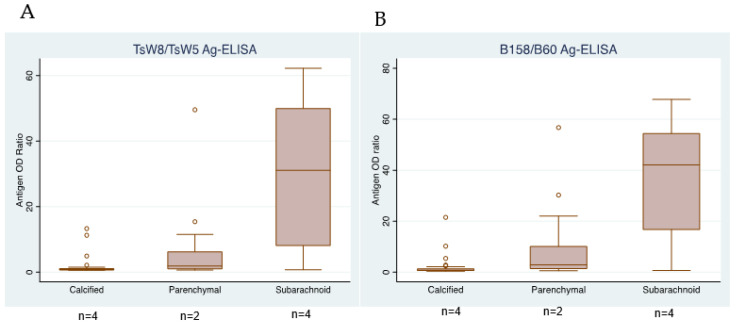
Boxplots showing the antigen OD ratios for each Ag-ELISA (**A**,**B**) in patients according to the type of NCC.

The proportions of antigen-positive subarachnoid NCC cases were 47/48 (97.8%) for both TsW8/TsW5 Ag-ELISAs and B158/B60 Ag-ELISAs. Similarly, the proportions of antigen-positive cases for both TsW8/TsW5 Ag-ELISAs and B158/B60 Ag-ELISAs in patients with viable parenchymal NCC were 19/24 (79.2%) and 21/24 (87.5%), respectively. In parenchymal NCC cases, these two discrepant samples showed a weak positive result (antigen ratios close to 1.5) in the B158/B60 Ag-ELISA, while for TsW8/TsW5 Ag-ELISA, the ratio was 0.8 for both samples. Similarly, the B158/B60 Ag-ELISA categorized as positive 18/41 (43.9%) samples from patients with calcified NCC, while the TsW8/TsW5 Ag-ELISA detected only 13/41 (31.7%). (Figure 1). In the same way as in the parenchymal discordances, the 5 discrepant samples in calcified NCC showed low antigen ratios varying between 1.2 and 2.5 versus 0.66 and 0.98 for TsW8/TsW5 Ag-ELISA. These disagreements in the results categorization were due to borderline or weak antigen levels. Discordances, defined as discrepancies in more than 30% of the assays between the antigen OD readings from both assays, were identified in 16/113, corresponding to 4 from calcified NCC, 4 from parenchymal NCC, and 8 from subarachnoid NCC. However, only 2 of these samples (belonging to calcified NCC) changed from a positive to negative result or vice versa, while the other 14 maintained their positive result.

The antigen ratios also showed a similar tendency according to the type of NCC in both Ag-ELISAs, with higher levels in patients with subarachnoid and viable parenchymal NCC, but lower levels in cases with calcified parenchymal NCC (Figure 1).

### 3.2. Agreement between Ag-ELISA Results

**Figure 2:**
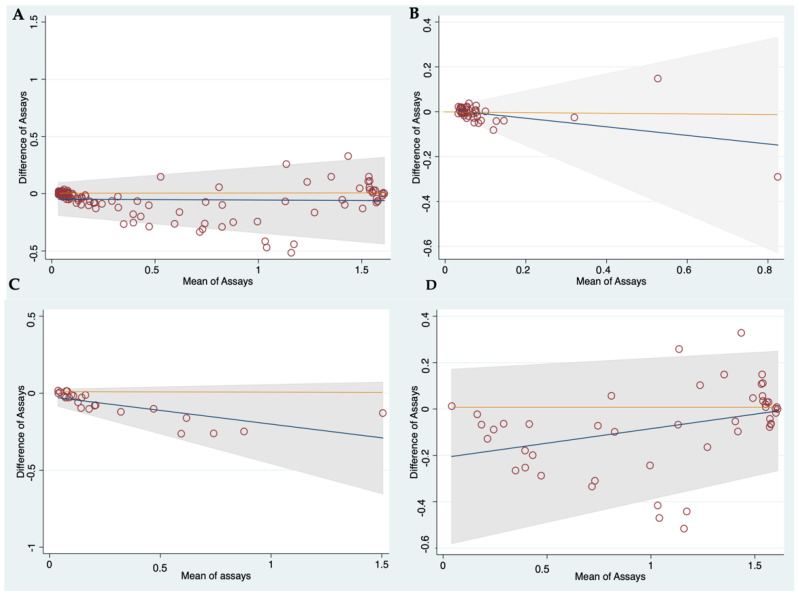
Bland–Altman plots accounting for trend showing the agreement between Ag-ELISAs for measuring circulating levels of parasite antigen (expressed as log-OD values). Y-axis (difference of measurements between assays); X-axis (mean of combined measurements). (**A**) All patients; (**B**) Calcified parenchymal NCC; (**C**) Viable parenchymal NCC; (**D**) Subarachnoid NCC.

The BA plot across all patients showed that 90.3% (102/113) of the paired log-OD differences between assays were within the LoA, with a regression line of paired differences (mean bias) very close to the line of perfect agreement (mean bias = −0.05 ± 0.1) (Figure 2A). Stratified analysis according to the type of NCC showed that 100% of the paired differences between assays were within the LoA in patients with viable parenchymal and calcified NCC, as were 89.6% (43/48) of the samples from patients with subarachnoid NCC (Figure 2A–D).

**Figure 3:**
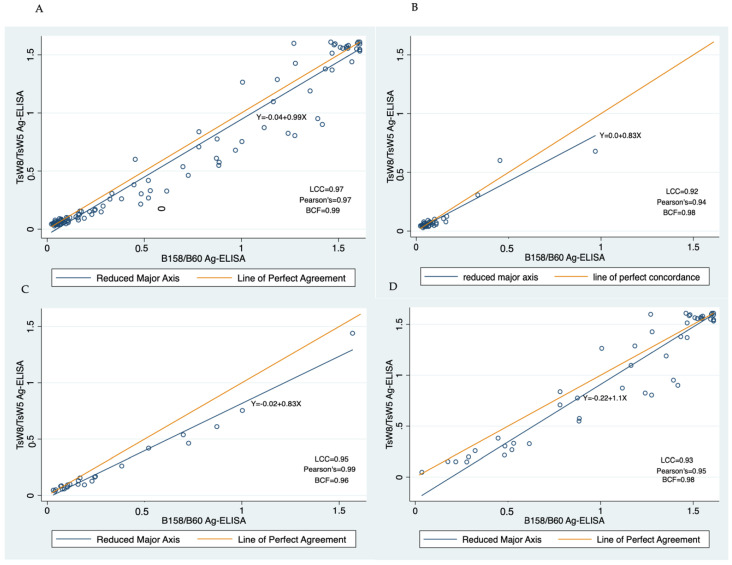
Scatter plots showing regression lines of log-OD values between Ag-ELISA’s with corresponding coefficients and 95% confidence intervals (blue line) versus the identity line (orange line). (**A**) All patients; (**B**) Calcified parenchymal NCC; (**C**) Viable parenchymal NCC; (**D**) Subarachnoid NCC.

The LCC of paired log-ODs also showed high agreement between Ag-ELISAs across all NCC patients (LCC = 0.97, 95% CI: 0.96–0.98). According to the type of NCC, the highest level of agreement between antigen assays was observed in patients with viable parenchymal NCC (LCC = 0.95; 95% CI: 0.92–0.98), followed by agreement between assays in patients with subarachnoid NCC (LCC = 0.93, 95% CI: 0.9–0.97) and calcified NCC (LCC = 0.92 (95% CI: 0.89–0.96, Table 2). Scatter plots also showed good agreement between the Ag-ELISAs across all patients and in all the subgroups of patients (Figure 3).

## 4. Discussion

In this study assessing 133 samples from NCC cases, the serum parasitic antigen levels detected by our TsW8/TsW5 Ag-ELISA were highly concordant with those obtained using another monoclonal antibody-based B158/B60 Ag-ELISA. There was a high agreement in detecting parenchymal viable and calcified lesions (100%), and likewise in detecting subarachnoid NCC (89.6%). There were some discordances (discrepancies in more than 30% of the antigen OD readings means between both assays), which could have resulted from the variability in each technique or, less likely, the identification of different antigenic epitopes. Likewise, the disagreements in the proportion of positive cases are due to antigen ratios influenced by the intrinsic parameters of each assay, such as cutoff values, causing discordances for borderline results, which are considered in the gray zone of ELISA assays. However, individuals with viable parasite infections are usually strongly positive on immunological tests. Strongly positive values thus have a high positive predictive value. On the contrary, weak positive results close to the cutoff point only infrequently represent a viable infection, resulting in a low positive predictive value, making borderline samples an ELISA gray zone poorly relevant in daily practice. Direct analyses of the log-OD values for agreement demonstrated a high concordance between both assays, according to Lin’s coefficient (overall agreement LCC = 0.97).

NCC is a pleomorphic disease with clinical manifestations influenced by the number, size, and location of lesions, as well as the human immune response [9,28]. While the definitive diagnosis relies on neuroimaging, immunodiagnostic tools play an important role in supporting the diagnosis [29,30]. While assays that detect antibody responses are generally more sensitive due to the amplification by the host immune system, antigen assays are more specific to viable infections since antigen levels correlate with the burden of viable parasitic lesions. Detection of circulating parasite antigens is useful to monitor the patient’s response to antiparasitic treatment, and it could also be used to screen endemic populations when aiming to detect individuals with a high burden of parenchymal cysts or with subarachnoid NCC, conditions with a high risk of future neurological complications [12,13,19,24,31]. A screening approach could facilitate early diagnosis and intervention, including referral for imaging and the specialized care as needed [32,33].

Antigen detection assays are based on the production of specific mAbs that can detect antigens, commonly adapted into an ELISA format. The Ag-ELISA is easy to perform and implement in non-specialized laboratories, and as a quantitative assay, antigen levels can be used as a proxy for the burden of viable infection and to monitor treatment efficacy [25]. So far, two assays have been well-described and tested in the literature, HP10/HP6 Ag-ELISA and B158/B60 Ag-ELISA. Both were developed to detect *Taenia saginata* cysticercosis, and only through inadvertent cross-reaction, they also allowed the diagnosis of *Taenia solium*. Protein characterization analysis demonstrated that the mAbs B158/B60 recognize a 65 kDa excretory/secretory product released from the cysticerci [34]. This assay has demonstrated good performance in detecting NCC cases (sensitivity 92% and specificity 98%) [24]. Although cross-reactions have been reported with other *Taenia* species in porcine cysticercosis [35], the specificity of the B158/B60 Ag-ELISA is extensively accepted for NCC diagnosis since *T. solium* is the principal *Taenia* that causes neuroparasitosis in humans.

Unlike the other available Ag-ELISAs, our test uses two mAbs produced against *T. solium* cysticerci components [26]. From 21 mAb clones directed against whole cyst (WA), vesicular content (VF), and excretory/secretory products (E/S) of *T. solium*, we identified eight clones capable of detecting circulating antigens in serum. The most promising pair of capture and detection mAbs (TsW8 and TsW5, respectively) was used in a sandwich ELISA format, as described here. A recent study has also proved an optimal performance of this assay in detecting urinary antigen in a rapid test format [36]. TsW8 and TsW5 recognized surface structures of the neck and cyst wall, and they have already demonstrated specificity against *E. granulosus* VF, *T. hydatigena* VF, and *F. hepatica* E/S [26].

As expected, both Ag-ELISA’s report lower antigen levels in calcified lesions (mean of antigen ratio = 0.8), whereas patients with viable parenchymal cysts were positive, with higher antigen ratios (mean of antigen ratios = 1.8 for TsW8/TsW5 Ag-ELISA and 2.9 for B158/B60 Ag-ELISA). Subarachnoid NCC is known to commonly saturate the detection limit of the assay (mean of antigen ratios = 31.1 for TsW8/TsW5 Ag-ELISA, and 42.1 for B158/B60 Ag-ELISA) [37,38], which is also concordant for both assays. Interestingly, four positive samples from calcified NCC presented high antigen levels for both assays, suggesting a possible undetected viable lesion in the imaging diagnosis. Indeed, further information about these NCC patients is needed for a correct interpretation of this serology result.

Our study has some drawbacks that need to be addressed. Our small sample size and lack of non-NCC samples did not allow assessing the assay’s sensitivity and specificity. Moreover, a better understanding of our new assay could be accomplished if we identify the antigen that our mAbs capture; unfortunately, our efforts to identify this target have been unsuccessful so far, apparently because of post-translational modifications.

Despite the above-mentioned limitations, our results demonstrate a robust assay performance, as demonstrated by a strong agreement with the B158/B60 Ag-ELISA in recognizing active infection in subarachnoid and viable parenchymal NCC cases. We were able to validate the use of our Ag-ELISA based on the comparable performance of mAbs TsW8/TsW5 and mAbs B158/B60 in detecting active lesions of NCC, providing a new available tool for antigen detection in NCC patients. Potential advantages of our new immunoassay would be the improvement of assays specific for *T. solium* and the multiple uses of our mAbs to develop low-cost techniques useful in rural areas. We look forward to the development of new studies to fully understand the nature of our *T. solium* mAbs and future studies to properly assess the test sensitivity and specificity, as well as to demonstrate its use for monitoring the treatment of patients with NCC.

## Figures and Tables

**Table 1:** Characteristics and antigen levels of study participants by type of NCC.

Characteristics	Total	Type of NCC			*p*
	(n = 113)	Calcified NCC	Parenchymal NCC (n = 24)	Subarachnoid NCC (n = 48)	
		(n = 41)			
Age (Years) *	43.4 ± 14.9	46.0 ± 15.5	33.5 ± 10.7	46.1 ± 14.3	0.009
Sex					
Male	56 (49.6)	21 (51.2)	9 (37.5)	26 (54.2)	0.397
Female	57 (50.4)	20 (48.8)	15 (62.5)	22 (45.8)	
Number of reactive EITB bands **	3 (3–7)	3 (2–3)	3 (2–5)	7 (5–7)	<0.001
TsW8/TsW5					
Ag-ELISA					
OD	0.17 (0.1–1.46)	0.06 (0.0–0.1)	0.12 (0.1–0.4)	2.4 (0.6–3.8)	<0.001
OD ratio ***	2.5 (0.9–21.8)	0.8 (0.7–1.1)	1.8 (1.0–6.2)	31.1 (8.1–50)	<0.001
Positive	79 (69.9)	13 (31.7)	19 (79.2)	47 (97.9)	<0.001
B158/B60					
Ag-ELISA					
OD	0.3 (0.1–2.3)	0.1 (0.0–0.1)	0.2 (0.1–0.6)	2.6 (0.1–3.6)	<0.001
OD ratio ***	4.6 (1.0–34.8)	0.8 (0.7–1.3)	2.9 (1.4–10.1)	42.1 (16.8–54.4)	<0.001
Positive	86 (76.1)	18 (43.9)	21 (87.5)	47 (97.9)	<0.001

* Mean ± standard error; ** Median (interquartile range); *** OD ratios in each Ag-ELISA were obtained using the corresponding negative controls.

**Table 2:** Lin’s concordance coefficients (with 95% CI), Pearson’s correlation coefficient, and bias correction factor (BCF) obtained from paired Ag-ELISA results in the total patient population and stratified by type of NCC.

Type of NCC	LCC (95% CI *)	*p*	Pearson’s *r*	BCF
Calcified NCC	0.92 (0.89–096)	<0.001	0.94	0.98
Viable parenchymal NCC	0.95 (0.92–0.98)	<0.001	0.99	0.96
Subarachnoid NCC	0.93 (0.9–0.97)	<0.001	0.95	0.98
TOTAL	0.97 (0.96–0.98)	<0.001	0.97	0.99

LCC (Lin’s concordance coefficient), Pearson’s *r* (Pearson’s correlation coefficient), BCF (Bias correction factor), * Confidence intervals estimated using the z-transform.

## Data Availability

Data are contained within the article or Supplementary Material.

## Supplementary Materials

The following supporting information can be downloaded at: https://www.mdpi.com/article/10.3390/pathogens12040566/s1, Table S1. Coefficients from Linear regression models to assess the agreement between Ag-ELISA’s for measuring antigen levels (expressed as log-OD values) in total study participants, and according to NCC type.
