# Supplementary material for: Consistent Measurement of Parasite-Specific Antigen Levels in Sera of Patients with Neurocysticercosis Using Two Different Monoclonal Antibody (mAb)-Based Enzyme-Linked Immunosorbent Assays
Source: Pathogens. 2023 Apr 6;12(4):566. doi: 10.3390/pathogens12040566 (PMC10143799; doi:10.3390/pathogens12040566)
Supplement: Supplementary file 1 [file pathogens-12-00566-s001.zip › pathogens-2301754-supplementary.pdf]

Supplementary Materials: Table S1. Coefficients from Linear regression models to assess the agreement between Ag-ELISA's for measuring antigen levels (expressed as log-OD values) in total study participants, and according to NCC type.

Variables	Crude Model				Multivariate Model *			
	$ß_0$ (95% CI)	*P*	$ß_1$ (95% CI)	*P***	$ß_0$ (95% CI)	*P*	$ß_1$ (95% CI)	*P***
Total (N = 113)	-0.01 (-0.07–0.04)	0.601	0.96 (0.90–1.02)	0.170	-0.03 (-0.13–0.07)	0.587	0.93 (0.85–1.01)	0.075
Parenchymal NCC (n = 24)	0.04 (-0.01–0.09)	0.096	1.21 (0.96–1.46)	0.104	0.04 (-0.11–0.18)	0.630	1.21 (0.98–1.43)	0.069
Calcified NCC (n = 41)	0.08 (0.04–0.11)	<0.001	0.21 (-0.27–0.69)	0.001	-0.09 (-0.33–0.15)	0.471	0.22 (-0.23–0.66)	0.001
Subarachnoid NCC (n = 48)	0.24 (0.14–0.34)	<0.001	0.82 (0.75–0.89)	<0.001	0.18 (-0.06–0.43)	0.152	0.84 (0.77–0.90)	<0.001

* Multivariate model included adjusted by age (years) and sex of participants, ** *P* for the hypothesis test that **$ß_1$ = 1).**
